# Transcriptome responses in alfalfa associated with tolerance to intensive animal grazing

**DOI:** 10.1038/srep19438

**Published:** 2016-01-14

**Authors:** Junjie Wang, Yan Zhao, Ian Ray, Mingzhou Song

**Affiliations:** 1 College of Ecology and Environmental Science, Inner Mongolia Agricultural University, Hohhot, China; 2 Department of Plant and Environmental Sciences, New Mexico State University, Las Cruces, NM, USA; 3 Department of Computer Science, New Mexico State University, Las Cruces, NM, USA

## Abstract

Tolerance of alfalfa (*Medicago sativa* L.) to animal grazing varies widely within the species. However, the molecular mechanisms influencing the grazing tolerant phenotype remain uncharacterized. The objective of this study was to identify genes and pathways that control grazing response in alfalfa. We analyzed whole-plant *de novo* transcriptomes from grazing tolerant and intolerant populations of *M. sativa* ssp. *falcata* subjected to grazing by sheep. Among the Gene Ontology terms which were identified as grazing responsive in the tolerant plants and differentially enriched between the tolerant and intolerant populations (both grazed), most were associated with the ribosome and translation-related activities, cell wall processes, and response to oxygen levels. Twenty-one grazing responsive pathways were identified that also exhibited differential expression between the tolerant and intolerant populations. These pathways were associated with secondary metabolite production, primary carbohydrate metabolic pathways, shikimate derivative dependent pathways, ribosomal subunit composition, hormone signaling, wound response, cell wall formation, and anti-oxidant defense. Sequence polymorphisms were detected among several differentially expressed homologous transcripts between the tolerant and intolerant populations. These differentially responsive genes and pathways constitute potential response mechanisms for grazing tolerance in alfalfa. They also provide potential targets for molecular breeding efforts to develop grazing-tolerant cultivars of alfalfa.

Plant tolerance to herbivory is a genetically controlled trait^1^, which has not been fully studied at the molecular level in alfalfa (*Medicago sativa* L). Although traditionally grown as a high nutritive value hay crop, this perennial forage legume can also be grown in monoculture or interseeded into temperate grassland pastures for direct grazing by livestock^2^. When interseeded into grass pastures, alfalfa can increase overall pasture biomass yield, overall forage crude protein content, palatability, digestibility, and consequently, animal performance^3^,^4^. However, a major challenge to utilizing alfalfa as a pasture legume is that frequent defoliation under continuous grazing ultimately reduces plant vigor and survival^4^,^5^,^6^,^7^,^8^.

Grazing resistance involves multiple mechanisms that impact the survival and growth of plants following grazing. Such mechanisms include avoidance strategies that reduce the probability and severity of defoliation, and tolerance mechanisms that promote growth following grazing^9^. Many morphological and physiological traits of alfalfa are associated with grazing resistance including deep-set crowns^10^, rhizome production^11^, subsurface shoot budding of crowns^12^, broad crowns^13^, prolific and nonsynchronous shoot budding^8^,^14^, extended periods of shoot bud initiation^15^, maintenance of leaf area^16^, maintenance of root carbohydrates^16^,^17^,^18^, disease resistance^7^,^19^ and pest resistance^20^. Efforts to develop grazing resistant alfalfa cultivars have been successful over the past 60 years with selection for rhizome production (i.e. the creeping root trait) and plant persistence under grazing representing the most common breeding strategies utilized^6^,^9^,^18^,^21^,^22^,^23^,^24^,^25^,^26^.

Underlying molecular mechanisms responsible for the phenotypic variation in alfalfa grazing tolerance (i.e. processes that promote plant growth following defoliation) are not well understood. However, it is known that many biotic and abiotic stresses, including herbivory, result in significant changes in the expression of genes involved in primary metabolism^27^. The production of reactive oxygen species in response to herbivory also triggers antioxidant defense and hormone signaling responses in many plant species^28^,^29^. Currently, a handful of high-throughput sequencing experiments have been performed to characterize the alfalfa transcriptome for a variety of processes. These include transcriptome analysis of glandular trichomes^30^, single-feature polymorphism discovery^31^, single nucleotide polymorphism (SNP) discovery^32^,^33^, and differential gene expression analysis associated with divergent cell wall composition^34^. No studies, however, have investigated the molecular profile of alfalfa in response to grazing stress. To address this gap, we developed two *Medicago sativa* ssp. *falcata* populations that differed in their grazing tolerance (i.e., the ability to generate forage biomass following continuous sheep grazing). We chose this alfalfa subspecies because it possesses traits crucial for survival under grazing, while such traits are less frequently observed in *M. sativa* ssp. *Sativa*^6^. To determine genes and pathways which may control tolerance to intensive grazing in alfalfa, RNA samples from grazed and ungrazed tolerant plants and grazed intolerant plants were evaluated in the current study using RNA sequencing and *de novo* transcriptome assembly. On the assembled transcriptome data, we performed differential gene expression analyses in the two contexts of gene ontology and functional pathway enrichment to overcome the low statistical power inherent in typical transcriptome experimental designs that possess a large number of transcripts but a small biological sample size. This comprehensive set of data has allowed us to identify differentially responsive processes and pathways associated with ribosomal subunit protein composition, cell wall formation, oxidative stress response, primary and secondary metabolism, translation, hormone signaling, defense signaling and response, and energy production. We also identified SNPs within five genes that were upregulated on these pathways. Therefore, these pathways and genes may play a key role in the alfalfa grazing tolerance response and provide targets for future molecular breeding efforts to improve grazing tolerance of alfalfa cultivars.

## Results

### Selection of grazing tolerant and intolerant alfalfa plants

Continuous sheep grazing of the *Medicago sativa* ssp. *falcata*, cultivar, ‘Hulunbeier’ over a three year period (2008–2010) near Bayantuohai, Inner Mongolia, China was utilized to identify 10 plants that recovered rapidly, and 10 plants that recovered poorly, after grazing pressure was removed. These two groups of plants were designated as MF200401 and MF200402, respectively. The grazing tolerant and intolerant phenotypes of MF200401 and MF200402 were visually confirmed when vegetative propagules of the 10 plants from each group were transplanted into a replicated spaced-plant nursery in fall 2010, and resubjected to continuous grazing throughout the 2011 and 2012 growing seasons. Phenotypic characterization of these plants in July 2012, prior to initiation of sheep grazing, indicated that the tolerant MF200401 plants possessed significantly greater shoot canopy area and basal plant diameter, taller shoots and a greater number of stems than the intolerant MF200402 plants (Table 1).

### *De novo* assembled transcriptomes of grazed and ungrazed alfalfa

Three whole-plant RNA bulks derived from the grazed and non-grazed MF200401 plants, and the grazed MF200402 plants, were utilized for transcriptome sequencing analysis to identify genes that were grazing responsive and differentially expressed between the tolerant and intolerant populations. Each whole-plant RNA bulk consisted of a balanced composite of RNA from leaf, stem, and root tissues from all 10 individuals of MF200401 or MF200402. RNA sequencing generated about 50 million raw reads from each of the three transcriptomes. The raw reads were assembled into 78,937 unique transcripts, about 90% of which were annotated by homology to known plant gene sequences. The assembled and annotated transcriptomes associated with the three alfalfa grazing treatments are summarized in Supplementary Table S1.

Gene repression and activation were observed in the tolerant MF200401 plants in response to grazing, where 39,738 transcripts (43%) exhibited differential expression (Supplementary Figure S1). In addition, 47,084 transcripts (54%) were differentially expressed between the grazed intolerant and tolerant populations. We subsequently focused our attention on genes that were grazing responsive in the tolerant MF200401 population, and also differentially expressed between the tolerant and intolerant populations which were both grazed.

### Ribosomal, cell wall, and oxygen-responsive activities are differentially impacted by grazing of tolerant and intolerant plants

Gene Ontology (GO) term enrichment analysis was used to initially characterize the alfalfa transcriptome in a global landscape of biological activities which were responsive to herbivory, and differentially expressed between the tolerant and intolerant populations. This process initially involved selecting enriched GO terms with a significant response (P ≤ 0.05) between the grazed and non-grazed tolerant plants. We subsequently removed those terms that were not differentially enriched (differential p-value > 0.05) between the tolerant and intolerant populations, both grazed. The remaining terms are summarized in Table 2 and contained 16 cellular component GO terms primarily related to the ribosome (11 terms) and cell wall (e.g “external encapsulating structure”, “extracellular region”, and “cell wall” terms), seven GO terms associated with diverse molecular function activities including ribosome structural constituents, pyruvate decarboxylase activity, oxidoreducatse activity, and oxygen binding, and four biological process GO terms affiliated with translation and oxygen response.

The GO terms associated with the ribosome and translation indicated that a number of translation-related activities were impacted by grazing of the tolerant MF200401 population. While we were unable to identify other published reports which have evaluated the impact of severe livestock grazing on ribosomal and translation-related processes, continuous defoliation is expected to influence primary metabolic pathways (e.g. carbohydrate and amino acid biosynthesis/metabolism) which would subsequently affect translational activities.

Regarding the GO terms associated with “pyruvate decarboxylase activity”, “oxygen binding”, “response to hypoxia”, “response to decreased oxygen levels”, and “response to oxygen levels”, such oxygen-influenced activities are consistent with molecular responses to a variety of abiotic and biotic stresses in plants, including insect herbivory^27^,^28^,^35^. These processes, in conjunction with others that impacted the cell wall, would have clearly been operational in the current study as continuous tissue and cell disruption was inflicted upon the plants by sheep grazing, trampling, and crushing of shoot tissues^36^. The presence of root RNAs in the transcriptomes from the grazed and nongrazed plots, and the impact of herbivore trampling on soil physical properties, also likely contributed to the detection of the oxygen-related GO terms. Although soil property differences in the grazed and nongrazed plots were not measured in our study, herbivore trampling at high stocking rates on medium textured soils (i.e. the silt loam soil on which the alfalfa plots were grown) has been documented to result in soil surface compaction (i.e. increased soil bulk density, increased soil mechanical impedance, and decreased soil porosity), which impedes soil oxygen diffusion rates^37^,^38^,^39^.

**Figure 1:**
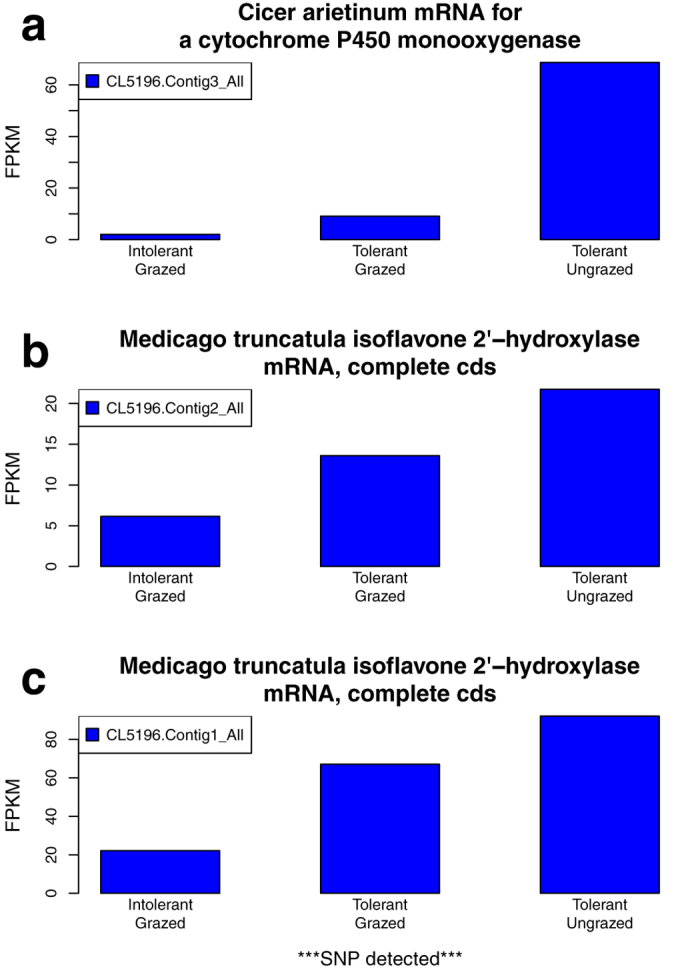
Grazing suppressed genes annotated with three oxygen-related GO terms, and differentially expressed between grazing tolerant and intolerant alfalfa plants. Analysis of the Gene Ontology terms “response to hypoxia,” “response to oxygen levels,” and “response to decreased oxygen levels” identified homologous transcripts of three genes that were suppressed in response to grazing of the tolerant plants. These genes were also expressed at an even lower level in the grazed intolerant plants. The vertical axis, fragments per kilobase of transcript per million mapped reads (FPKM), represents the normalized read abundance of homologous transcripts of a given gene. Homologous transcripts from the isoflavone 2′−hydroxylase (Contig1_All) gene, also possessed SNPs between the tolerant and intolerant plants.

Given that the tolerant and intolerant populations exhibited differential grazing responses for three oxygen-related GO terms, we conducted two additional analyses to identify transcripts that contributed to these outcomes. In the first “suppression” analysis we identified three candidate genes (Fig. 1) associated with these oxygen-related terms, that were suppressed in response to grazing of the tolerant plants, and which also possessed fewer transcripts in the intolerant grazed plants than the tolerant grazed plants. These three genes belonged to the cytochrome P450 monooxygenase (CYP) class of enzymes, including the *Cicer arietinum* CYP (Fig. 1a) and two *Medicago* isoflavone 2′-hydroxylases (Fig. 1b,c). BLASTx analysis of the *C. arietinum* CYP transcript against nonredundant protein databases indicated that its best match was with isoflavone 2′-hydroxylase of *M. truncatula* (87% identity, E-value 4e-59). We also detected a SNP in the 3′ UTR for the isoflavone 2′-hydroxylase CL5196.Contig1_All transcript between the tolerant and intolerant populations (Table 3). While the tissue source of these three genes is unknown in our study, Liu *et al*.^40^ reported that isoflavone 2′-hydroxylase (MtCYP81E) is predominately expressed in *Medicago* roots. If each of these three oxidoreductases were derived from root tissues, impaired root metabolic processes in oxygen-limited soil environments, from which the grazed MF200401 and MF200402 roots were isolated, could potentially account for reduced expression of these genes relative to the nongrazed MF200401 control. Given the key role of isoflavonoids in promoting root nodule formation for biological nitrogen fixation^41^ and synthesis of phytoalexins for pathogen defense response^42^, greater levels of expression observed for these candidate genes in the tolerant MF200401 population could contribute towards alfalfa productivity under grazed conditions.

**Figure 2:**
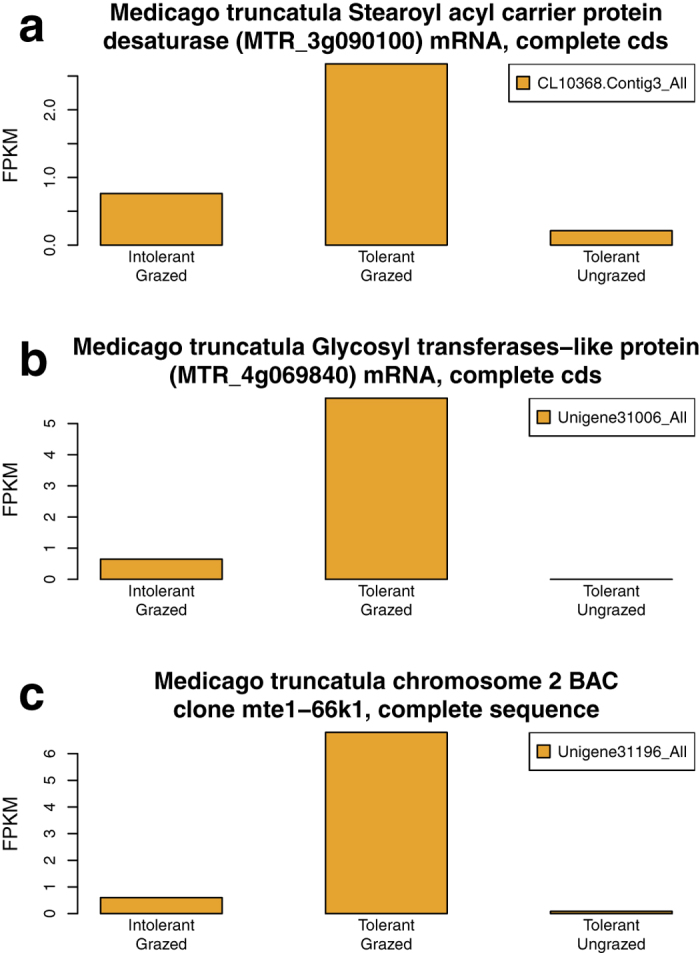
Grazing upregulated genes annotated with three oxygen-related GO terms and differentially expressed between tolerant and intolerant alfalfa plants. Analysis of the Gene Ontology terms “response to hypoxia,” “response to oxygen levels,” and “response to decreased oxygen levels” identified homologous transcripts of three genes that were consistently upregulated in response to grazing of the tolerant plants. These genes were also more strongly expressed in the tolerant versus intolerant plants, which were both grazed. The vertical axis, fragments per kilobase of transcript per million mapped reads (FPKM), represents the normalized read abundance of homologous transcripts of a given gene.

In the second “upregulation” analysis, we identified three candidate genes associated with the oxygen-related GO terms that were upregulated (Fig. 2) in response to grazing of the tolerant plants, and which were also more strongly expressed in the tolerant grazed plants than the intolerant grazed plants. Two of these genes possessed functional annotations including a stearoyl acyl-carrier protein desaturase (Fig. 2a) and a glycosyltransferase-like protein (Fig. 2b). Branco-Price *et al*.^43^ and Mustroph *et al*.^44^ reported that expression of a stearoyl acyl-carrier protein desaturase increased significantly in response to oxygen deprivation in shoots and roots of *Arabidposis*. Members of this gene family regulate fatty acid metabolism^45^, and catalyze the first step in the conversion of stearic acid to linolenic acid, a precursor for the fatty acid-derived signaling molecule, jasmoic acid^46^. This compound subsequently plays a key role in modulating crosstalk between different defense signaling pathways including activation of wounding response against insect herbivory in *Arabidopsis*^47^,^48^. Stearoyl acyl-carrier protein desaturases have also been associated with the cell wall matrix^49^ and shoot apical meristem organization^50^. While the specific role of the glycosyltransferase-like protein is unknown, many members of this large protein family in *Arabidopsis* and cotton (*Gossypium hirsutum*) are responsive to one or more abiotic and biotic stresses (including oxidative stress, hypoxia and wounding), where they appear to modulate plant defense response including jasmonic acid and salicylic acid signaling crosstalk^51^,^52^,^53^. Members of this family also play key roles in cell wall biosynthesis^54^,^55^, and generation of secondary metabolites, including terpenoid-based compounds, that influence plant architecture and defense^56^,^57^,^58^.

### Molecular pathways differentially responsive to grazing between tolerant and intolerant plants

**Figure 3:**
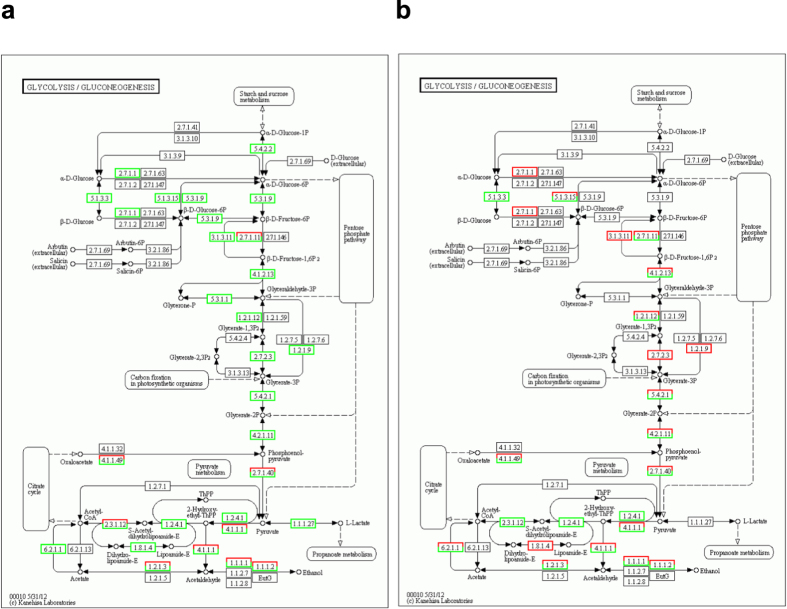
Impact of grazing on the glycolysis/gluconeogenesis pathway in grazing tolerant and intolerant alfalfa plants. The pathway model was obtained from KEGG Pathway^59^. **(a)** Identification of grazing responsive genes in the tolerant population. Green boxes surrounding EC identifiers represent genes with greater transcript abundance in the ungrazed versus grazed tolerant plants. Red boxes identify genes with transcripts that were more abundant in the grazed versus ungrazed tolerant plants. Boxes with mixed colors indicate both under-expression and over-expression of different transcripts of the same gene. Seventeen genes were consistently suppressed, while eight genes exhibited variable expression. Results suggest a strong suppression of this pathway in response to grazing. (**b**) Identification of 21 grazing responsive genes from (**a**) that were differentially expressed between tolerant and intolerant plants, both grazed. Green boxes identify genes with greater transcript abundance in the tolerant versus intolerant plants. Red boxes identify genes with greater transcript abundance in the intolerant versus tolerant plants. Boxes with mixed colors are as described above. Three and five genes were more abundantly expressed in the grazed tolerant and intolerant populations, respectively. Thirteen other genes possessed different homologous transcripts whose abundance varied between the tolerant in tolerant plants. Results suggest that expression of grazing responsive genes was highly variable between tolerant and intolerant plants, both of which were grazed.

To examine potential responses of metabolic, cellular, and other molecular processes to grazing stress, we performed enrichment analysis of 127 KEGG pathways that are related to *Medicago*^59^. We first inspected each pathway for enrichment of homologous transcripts of pathway genes between the grazed and non-grazed MF200401 tolerant plants and marked each pathway with a responsive p-value for statistical significance and a responsive q-value for false discovery rate. We then examined each pathway for differential enrichment of homologous transcripts between the tolerant and intolerant plants subjected to grazing and determined the differential p-value and q-value associated with each pathway. Based on a p-value threshold of 0.05, we identified 21 pathways that were both responsive to grazing and exhibited differential gene regulation between the grazed tolerant and intolerant populations (Table 4). One of these pathways (glycolysis/gluconeogenesis) is highlighted in Fig. 3 to illustrate the general process used for pathway enrichment analysis. Analysis of the transcriptomes associated with the grazed and ungrazed MF200401 population initially identified 25 grazing responsive genes, the majority of which (17) were suppressed under intensive grazing (Fig. 3a). Twenty-one of these genes were differentially expressed between the tolerant and intolerant populations, both grazed (Fig. 3b). These included three genes that were more abundantly transcribed in the grazed tolerant plants, five genes that were more abundantly expressed in the grazed intolerant plants, and 13 genes which possessed different homologous transcripts whose abundance varied between the tolerant and intolerant plants that were grazed. Among the latter group of genes, three enzymes (pyruvate decarboxylase, EC 4.1.1.1; alcohol dehydrogenase, EC 1.1.1.1; and alcohol dehydrogenase NADP +, EC 1.1.1.2) involved in anaerobic fermentation processes were consistent with the GO term enrichment analysis results associated with “pyruvate decarboxylase”, “hypoxia” and “decreased oxygen levels”.

Concerning biochemical pathways that were identified, we noted that most could be assigned to three general categories: (1) primary carbohydrate metabolism including pentose and glucuronate interconversions, starch and sucrose metabolism, glycolysis and gluconeogenesis, and galactose metabolism; (2) secondary metabolic pathways dependent upon biosynthesis of terpenoids including zeatin biosynthesis, diterpenoid biosynthesis, sesquiterpenoid and triterpenoid biosynthesis, carotenoid biosynthesis, and limonene and pinene degradation; and (3) shikimate derivative dependent pathways including phenylpropanoid biosynthesis, stilbenoid, diarylheptanoid and gingerol biosynthesis, cyanoamino acid metabolism, isoflavonoid biosynthesis, flavonoid biosynthesis, phenylalanine metabolism, and flavone and flavonol biosynthesis.

### Identification of grazing tolerance candidate genes: Herbivory suppressed genes

**Figure 4:**
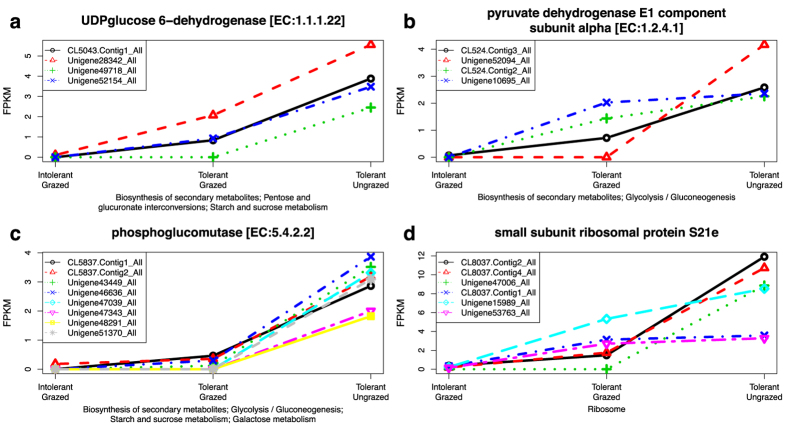
Grazing suppressed genes from grazing responsive molecular pathways that were differentially expressed between grazing tolerant and intolerant alfalfa plants. Homologous transcripts of 9 genes were suppressed by grazing in the tolerant population and were expressed at lower levels in the intolerant versus the tolerant plants both of which were grazed. Representative expression profiles of four genes from four different pathways are provided in (**a–d**). Complete results are available in Supplemental Figure 2S. The horizontal axis identifies the three transcriptomes that were evaluated: tolerant plants not grazed, tolerant plants grazed, and intolerant plants grazed. The vertical axis, fragments per kilobase of transcript per million mapped reads (FPKM), represents normalized read abundance of homologous transcripts of a given gene.

To identify genes that may actively participate in the grazing tolerance response, we further examined the three alfalfa transcriptomes to detect genes associated with the 21 grazing responsive pathways (Table 4) that were more abundantly expressed in the grazed tolerant plants versus the grazed intolerant plants. Two general categories of transcripts were identified: those which were suppressed (this subsection) or upregulated (next subsection) by grazing. Nine genes represented by 53 transcript isoforms, which were suppressed by grazing in the tolerant population, and which possessed even fewer transcripts in the grazed intolerant population, were discovered in this process (Fig. 4 and Supplementary Figure S2). Six of these genes were generally associated with secondary metabolism (pathway ko01110), however, each is also known to function in primary carbohydrate metabolic processes. For instance, UDP-glucose 6-dehydrogenase (Fig. 4a), pyruvate dehydrogenase E1 component (Fig. 4b), phosphoglucomutase (Fig. 4c), as well as, fructose-bisphosphate aldolase class II and UDP-sugar pyrophosphorylase (Supplemental Figures S2d and S2f, respectively) were clearly affiliated with four grazing responsive pathways including pentose and glucocuronate interconversions (pathway ko00040), starch and sucrose metabolism (pathway ko00500), glycolysis/gluconeogenesis (pathway ko00010), and galactose metabolism (pathway ko00052). Two enzymes associated with the above pathways, UDP-glucose 6-dehydrogenase and UDP-sugar pyrophosphorylase, have also been implicated in cell wall biosynthesis in *Arabiodpsis* and maize^55^,^60^,^61^,^62^,^63^. In addition, fructose-bisphosphate aldolase class II and mannose-1-phosphate guanylyltransferase (Supplemental Figure S2a) play key roles in fructose and mannose metabolism, although that pathway (ko00051) was not specifically identified in the pathway enrichment analysis. Three ribosomal subunit proteins, S23e (Fig S2g), L17e (Fig S2h), and S21e (Fig S2i) were also identified.

Collectively, these results indicated that two-thirds of the grazing suppressed genes could be associated primary carbohydrate metabolic pathways. Such results are reasonable given that frequent defoliation under continuous grazing removes source tissues, which subsequently reduces carbon assimilation, carbohydrate metabolism and storage. Consequently, reduced availability of energy resources could negatively impact other metabolic pathways, including ribosomal and translation-related processes as demonstrated by a reduction in transcript abundance for three ribosomal subunit proteins in the grazed plants. While grazing repressed the above genes in both populations, our analysis identified those genes which were impacted less severely in the tolerant MF200401 population. Such results indicate that grazing tolerance may be associated with a plant’s ability to maintain greater levels of functionality for primary carbohydrate metabolic processes and protein synthesis. These outcomes are consistent with Smith *et al*.^18^ who observed higher levels of total nonstructural root carbohydrates in grazing tolerant versus intolerant alfalfa cultivars subjected to continuous grazing by beef cattle. Volenec *et al*.^64^ and Ourry *et al*.^65^ have also demonstrated that defoliation tolerance is strongly impacted by the deposition of amino acids and vegetative storage proteins in roots and stem bases of diverse forage species (including alfalfa), whereby they provide an important source of N reserves that can be rapidly remobilized to developing leaves and shoots during forage regrowth.

### Identification of grazing tolerance candidate genes: Herbivory upregulated genes

**Figure 5:**
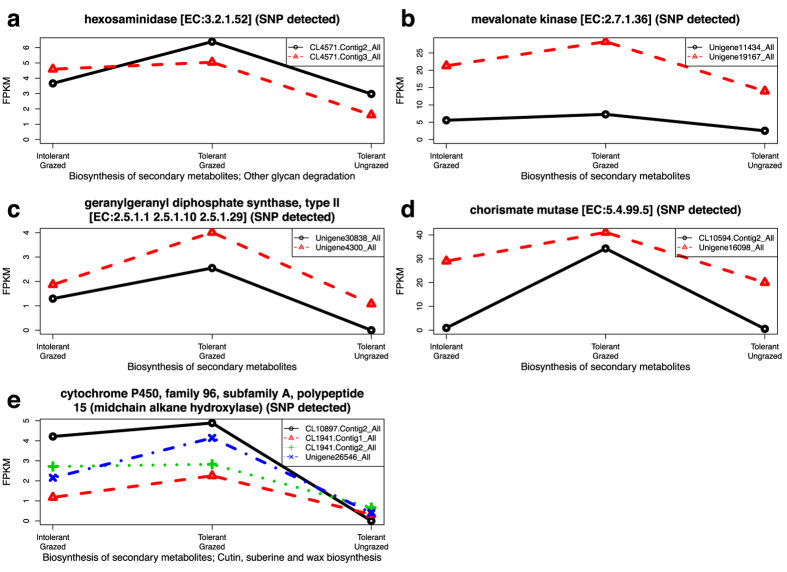
Grazing upregulated genes from grazing responsive pathways that were differentially expressed between grazing tolerant and intolerant alfalfa plants. Homologous transcripts of five genes were upregulated by grazing in the tolerant population, and were expressed at higher levels in the tolerant versus the intolerant plants which were both grazed. Pathways associated with each gene are also provided. The horizontal axis identifies three transcriptomes that were evaluated: tolerant plants not grazed, tolerant plants grazed, and intolerant plants grazed. The vertical axis, fragments per kilobase of transcript per million mapped reads (FPKM), represents normalized read abundance of homologous transcripts of a given gene. At least one SNP was detected between homologous transcripts of the tolerant and intolerant plants for each of these genes.

**Figure 6:**
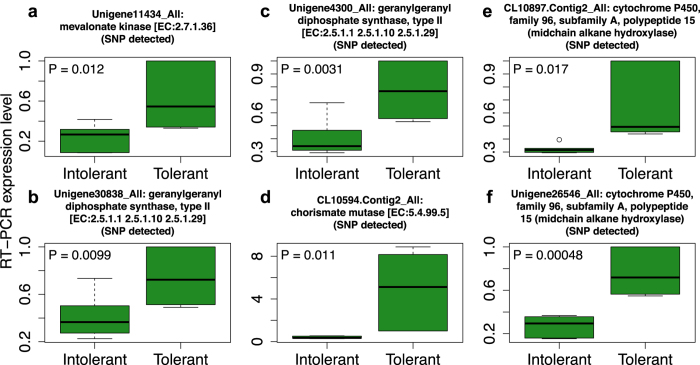
Grazing-upregulated genes that were expressed more abundantly in the tolerant versus intolerant alfalfa plants were independently confirmed by RT-PCR. Twelve transcripts from five grazing-upregulated transcript isoform groups were selected for RT-PCR expression quantification to validate transcriptome analysis results. Three biological replicates consisting of three tolerant and three intolerant alfalfa plants, all of which were grazed, and two technical replicates of each plant, were used in the RT-PCR analysis. Among the transcripts evaluated, six demonstrated significant expression level differences between the tolerant and intolerant alfalfa plants. The expression profiles of those six transcripts are provided in the box plots above, where transcript identities and their encoded proteins are given as plot titles. (**a**) One transcript encoding mevalonate kinase. (**b,c**) Two transcript isoforms encoding geranylgeranyl diphosphate synthase. (**d**) One transcript encoding chorismate mutase. (**e,f**) Two transcript isoforms encoding a cytochrome P450 midchain alkane hydroxylase. Box plots indicate the median and range of gene expression observed for each transcript among the tolerant and intolerant plants, and the *P*-value associated with the *t* test for differential expression between the tolerant and intolerant alfalfa plants. The circle in (**e**) indicates an outlier. For each of the six transcripts, expression levels were greater in the grazed tolerant versus the grazed intolerant plants, which is consistent with the transcriptome analysis results. Complete results of all 12 transcripts are available in Supplemental Figure S3.

We also searched the 21 differentially responsive pathways to identify genes that were consistently upregulated by grazing of the tolerant MF200401 plants, but which were expressed at lower levels in the grazed intolerant MF200402 plants. Five genes represented by 12 transcript isoforms were identified (Fig. 5) including hexoamindase (Fig. 5a), mevalonate kinase (Fig. 5b), geranylgeranyl diphosphate synthase (Fig. 5c), chorismate mutase (Fig. 5d), and cytochrome P450, family 96, subfamily A, polypeptide 15 (CYP96A15) (Fig. 5e). An independent RT-PCR quantification of all 12 transcripts (Fig. 6 and Supplementary Figure S3) from these five genes validated most of the transcriptome analysis results. We specifically focused on quantifying expression levels of these five upregulated genes for our validation analysis because sequence variants (i.e. SNPs), as described later in this report, were identified among the transcripts derived from each of these genes. The RT-PCR confirmed that six transcripts of four genes were expressed at significantly (P < 0.05) higher levels in the grazed MF200401 (tolerant) versus the grazed MF200402 (intolerant) population (Figs 5 and 6). In addition, RT-PCR results suggested that four of the remaining six transcripts were also more abundant in the tolerant versus the intolerant accession (Supplementary Figure S3). Although the specific contribution of these transcripts towards the alfalfa grazing response is currently unknown, they are known to play vital roles in growth, development, and abiotic and biotic stress response in a variety of organisms as summarized below.

Hexosaminidase (Fig. 5a and Supplementary Figure S3k,l), associated with pathway ko00511 (other glycan degradation), plays a role as a dynamic regulator of protein activity^66^. This protein participates in the nutrient responsive hexosaminidase signaling pathway through the removal of a O-GlcNAc monosaccharide from serine and threonine residues of many nuclear and cytosolic proteins^66^,^67^. Both the addition and removal of an O-GlcNAc moiety by O-GlcNAc transferase (OGT) and O-GlcNAcase (hexosaminidase), respectively, dramatically alters the function of target proteins in animals and plants. Among the many plant proteins affected by this process are those involved in the gibberellin signaling pathway^68^,^69^ which influences shoot growth and development, cytokinin catabolism and drought response^70^. The potential involvement of this gene in differentially regulating hormone activity in response to grazing^27^ is consistent with the activation of two other genes (mevalonate kinase and geranylgeranyl diphosphate synthase) that participate in the biosynthesis of giberillins and cytokinins, as described below.

Mevalonate kinase (Figs 5b and 6a), which was associated with multiple pathways involved in the biosynthesis of secondary metabolites (e.g. pathway ko01110, Table 4), takes part in the synthesis of plant isoprenoids. These compounds provide terpenoid backbones for the synthesis of many products including: the cytokinin, zeatin (pathway ko00908); growth promoting gibberellins and brassinosteroids (pathway ko00909 and pathway ko00904); and carotenoid pigments, and carotenoid-derived hormones such as abscisic acid and strignolactones (pathway ko00906)^57^,^71^,^72^,^73^. The role of mevalonate kinase in plant growth and development is illustrated by work of Tang and Newton^74^ who reported that activity for this enzyme peaked during callus induction and the formation of shoots and roots in tissue culture regenerated white pine (*Pinus strobus*). In addition, overexpression of this kinase in tobacco induced cytokinin synthesis^75^,^76^ and promoted shoot and root cell division. This protein has also been implicated as playing a role in the conversion of maize etioplasts to chloroplasts^77^.

Geranylgeranyl diphosphate (GGPP) synthase type II (Figs 5c and 6b,c) is another key enzyme involved in the synthesis of terpenoid backbones and their derivatives. This synthase has been reported to be up-regulated in response to insect-herbivory^78^. GGPP synthase also induces biosynthesis of ubiquinone (coenzyme Q) for cellular respiratory processes associated with oxidative phosphorylation, where energy production occurs downstream of glycolysis and consumes pyruvate in the mitochondria to create ATP^79^. The grazing induced expression of transcripts for GGPP synthase, and mevalonate kinase, are consistent with the enrichment of several GO terms (Table 2) affiliated with pyruvate decarboxylase activity, and oxygen-related responses which may also involve the generation of active oxygen species in response to herbivory^27^,^35^. These results are also consistent with other reports which have identified a variety of terpenoid-based compounds produced in response to insect herbivory^80^,^81^, and which influence plant architecture and defense^56^,^57^,^58^.

Chorismate mutase (Figs 5d and 6d) catalyzes the conversion of chorismate to prephenate^82^ in the shikimate pathway, and is widely recognized for its role in phenylalanine, tyrosine and tryptophan biosynthesis and metabolism (pathway ko00360, Table 4). Products of this pathway are extensively utilized in phenylpropanoid metabolism (pathway ko00940) including the synthesis of stilbenoids, diarylheptanoids and gingerols (pathway ko00945), cutins and suberins (pathway ko00073), isoflavonoids (pathway ko00943), flavonoids (pathway ko00941) and their flavone and flavonol derivatives (pathway ko00944). This pathway is responsive to light and wounding^83^, including wounding associated with insect herbivory^81^. Activation of this gene, as well as, mevalonate kinase and GGPP synthase, could imply that grazing tolerant plants may be jointly utilizing products from the shikimate and terpenoid backbone pathways, to provide precursors for synthesizing ubiquione (an electron carrier for the mitchondrial electron transport chain, see GGPP synthase discussion above), and other terpenoid-quinones such as plastoquinones (involved in photosynthetic electron transport), tocopherols and phylloquinones^84^.

The cytochrome P450, family 96, subfamily A, polypeptide 15 (CYP96A15, midchain alkane hydroxylase, Figs 5e and 6e,f) participates in stem cuticular wax biosynthesis in *Arabidopsis*^85^ (Table 4, pathway ko00073). Activation of genes in the wax biosynthesis pathway have been shown to contribute to *Arabidopsis* drought tolerance^86^ and may protect alfalfa plants during grazing by reducing tissue desiccation processes resulting from herbivory and trampling.

### Sequence polymorphisms within grazing tolerance candidate genes

Based on the grazing responsive candidate genes that were collectively identified in the GO term and pathway enrichment analyses, we subsequently searched for SNPs among their transcripts. These included eight grazing-upregulated genes in the tolerant plants (Figs 2 and 5) and 12 genes that were suppressed in response to grazing of the tolerant plants (Figs 1 and 4 and Supplementary Figure S2). Both groups of genes possessed a greater abundance of transcripts in the grazed tolerant population as compared to the grazed intolerant population. Unique homologous transcript polymorphisms between the tolerant and intolerant alfalfa populations were detected for five grazing-upregulated genes affiliated with the pathway enrichment analysis, and one grazing-suppressed gene affiliated with the GO term enrichment analysis. The specific polymorphisms and their location within each of the homologous transcript assemblies are presented in Tables 3 and 5. For the grazing-upregulated genes these polymorphisms included: one hexosaminidase SNP resulting in a synonymous amino acid substitution; two mevalonate kinase SNPs with one each in the 5′ and 3′ UTR; one GGPP synthase SNP resulting in a non-synonymous amino acid substitution; two chorismate mutase SNPs resulting in one synonymous and one non-synonymous amino acid substitution; and one CYP96A15 SNP resulting in a synonymous amino acid substitution. As previously discussed, we also detected a 3′UTR SNP between homologous transcripts of the isoflavone 2′-hydroxylase gene which was suppressed in response to grazing. These results suggest that expression of alternative alleles or gene family members in the tolerant and intolerant plants, and post-transcriptional regulation events associated with 5′ and 3′ UTR sequence alterations, and altered protein function resulting from nonsynonymous amino acid substitutions, may be contributing towards the grazing tolerance phenotype in alfalfa.

## Discussion

As described in our introductory comments, many morphological and physiological traits of alfalfa are associated with grazing tolerance. The transcriptome analysis results of this study provide initial insight into the molecular mechanisms that may drive these phenotypes. In this regard, we identified a number of genes involved in the glycolysis, gluconeogenesis, fructose, mannose, galactose, sucrose and starch, metabolic pathways, which decreased significantly in the tolerant alfalfa plants as a consequence of grazing. These results are consistent with the expectation that continuous removal and damage of photosynthetic tissues by intensive animal grazing and trampling reduces carbon assimilation capabilities. Limited availability of carbon skeletons would subsequently impact many other processes including those associated with the ribosome, protein synthesis, and secondary metabolism.

Genes contributing to cell wall, phenylpropanoid, and isoflavonoid synthesis were also identified as being negatively impacted by grazing in the tolerant population. Concerning the synthesis of isoflavonoids, reduced expression of three isoflavone 2′-hydroxylase genes, which are primarily expressed in *Medicago* roots, was associated with the GO biological process terms “response to hypoxia” and “response to reduced oxygen levels”. These outcomes suggest that reduced soil oxygen diffusion rates resulting from animal-traffic-induced soil surface compaction may significantly impact alfalfa root metabolic processes; particularly those involved in the synthesis of isoflavonoids which promote root nodule formation for biological nitrogen fixation and synthesis of phytoalexins for pathogen defense response.

Although the above genes were suppressed in the tolerant plants in response to grazing, our analysis indicated that these same genes were expressed at higher levels in the grazed tolerant MF200401 population as compared to the grazed intolerant MF200402 population. These observations suggest that a plant’s ability to maintain greater levels of functionality for some metabolic processes involved in translation, carbon assimilation and metabolism, pathogen defense response, and root-nodule formation may significantly contribute towards alfalfa grazing tolerance. Such outcomes agree with reports that defoliation tolerance in alfalfa is influenced by a plant’s ability to store root carbohydrates and N reserves (e.g. protein and amino acids) that can be rapidly remobilized to drive forage regrowth^18^,^64^,^65^.

Eight additional genes were identified as being upregulated in the tolerant plants in response to grazing. These genes were also expressed at higher levels in the tolerant versus intolerant populations which were both subjected to grazing. These differential responses influenced cellular structure, molecular function and biological processes, as detected in the GO term analysis (Table 1), which were driven through a series of altered pathway activities as reported in Table 4. We speculate that four of these eight enzymes (hexoaminidase, mevalonate kinase, geranylgeranyl diphosphate synthase, and chorismate mutase) may potentially interact with each other through a network involving hexosaminidase and giberillin signaling, terpenoid backbone biosynthesis, chorismate synthesis, and ubiquinone and terpenoid-quinone biosynthesis. These interactions could significantly alter plant developmental and respiratory processes in response to grazing. Enhancement of such capabilities, in conjunction with increased wax biosynthesis to reduce tissue desiccation caused by herbivory and trampling as implicated by grazing activation of CYP96A15, appear to control critical traits that contribute towards the grazing tolerant phenotype of alfalfa population MF200401. Activation of two additional genes (stearoyl acyl-carrier protein desaturase and a glycosyl transferase-like protein) also suggests that tolerant plants possess enhanced herbivory and oxidative defense signaling and response capabilities involving biosynthesis of jasmoic acid, cell walls, and secondary metabolites when subjected to severe grazing stress.

Single nucleotide polymorphisms were detected in the transcripts of one grazing suppressed gene and five grazing upregulated genes. Each of these genes was expressed more abundantly in the tolerant MF200401 population than the intolerant MF200402 population, both of which were grazed. Among the eight SNPs identified, three were located in 5′ or 3′ UTRs and two resulted in nonsynonymous amino acid substitutions. Each of these changes could influence either post-transcriptional regulation or protein function. These results suggest that expression of alternative alleles or gene family members for these particular loci may play critically important molecular roles in governing differential response mechanisms which confer grazing tolerance to *M. sativa* ssp. *falcata*.

Although some inconsistencies were observed between the outcomes of the transcriptome and quantitative RT-PCR analyses, such results likely reflect the following: (1) alfalfa is an allogamous autotetraploid, (2) grazing tolerance appears to be a quantitative trait, and (3) RNA was bulked over 10 individuals from each of the grazing tolerant and intolerant populations for the transcriptome analysis, while RNA from three separate individuals of each population was evaluated by RT-PCR. Consequently, each of the 10 plants comprising each population was genetically heterogeneous, and the transcriptome analysis results represent the average gene expression levels over all plants. However, gene expression levels between the genetically distinct individuals used in the RT-PCR analysis are expected to vary to a greater extent. Such variation can be attributed to multiple loci controlling the grazing tolerance phenotype, where any given tolerant plant may possess desirable alleles at many, but not necessarily all, of the target loci. Similarly, intolerant plants may possess undesirable alleles at many, by not necessarily all, of the target loci. Given that expression levels for >80% of the transcripts evaluated by RT-PCR were in agreement with the transcriptome analysis results, we conclude that the differentially expressed genes and pathways identified in our study provide unique insight into potential molecular mechanisms responsible for the grazing tolerance phenotype in alfalfa.

## Conclusions

By studying the alfalfa transcriptome in response to grazing, we identified differentially responsive processes and pathways associated with ribosomal subunit protein composition, cell wall formation, oxidative stress response, primary and secondary metabolism, translation, hormone signaling, and defense signaling and response. In these responsive pathways, 12 grazing suppressed genes and eight grazing upregulated genes were detected from the tolerant MF200401 plants. Each of these 20 genes were expressed at significantly higher levels in the grazed tolerant populations, MF200401, suggesting that they may contribute towards molecular mechanisms conferring grazing tolerance to *M. sativa* ssp. *falcata*. In addition, single nucleotide polymorphisms between the tolerant and intolerant populations were detected in the transcripts of six of these genes indicating that expression of alternative alleles, or gene family members, may also play key roles in the grazing response process. Therefore, these pathways and genes may provide targets for future molecular breeding efforts to improve grazing persistence of alfalfa cultivars.

## Methods

### Plant material

To initially identify grazing tolerant and intolerant alfalfa plants for this study, a 0.45 ha pasture of the *Medicago sativa* ssp. *falcata* cultivar ‘Hulunbeier’ was planted at a seeding rate of 17 kg ha^−1^ in May 2008 at the Ewenke Banner Forage Research Station, Bayantuohai, Inner Mongolia, China. The soil texture at this site was a well-drained silt loam, highly favorable for alfalfa growth. Continuous heavy grazing with sheep was subsequently imposed upon these plants throughout the growing seasons of 2008, 2009, and 2010, such that plant heights remained at ≤8 cm. The sheep were removed on 31 August 2010 and the overall vigor of the surviving plants was visually evaluated two weeks later, on September 14, 2010. Ten plants which possessed the highest vigor scores were identified as grazing tolerant and designated as population MF200401 (Supplementary Figure S4a). Ten plants which possessed the lowest vigor scores were identified as grazing intolerant and were designated as population MF200402 (Supplementary Figure S4b). The 20 plants representing the two populations were then dug from the field and the crown of each plant was divided into seven equal portions. These clonal propagules were then transplanted on 30 cm centers in a nearby field using a randomized complete block experimental design with seven blocks, where the ten propagules of both populations were randomly arranged within each block.

To validate the tolerant and intolerant phenotypes of populations MF200401 and MF200402, respectively, and to provide tissue samples suitable for identifying changes in gene expression associated with differential tolerance to grazing, the transplanted propagules were resubjected to continuous sheep grazing during 2011. Prior to initiating the 2011 grazing treatment, however, one block was randomly designated as an ungrazed control and surrounded by a fence to prevent grazing. Grazing intensity and duration for the remaining six blocks was conducted in a manner similar to that described for the 2008 – 2010 study. The sheep were removed on 31 August 2011 and the overall vigor of the surviving plants was visually evaluated on 14 September. In 2012, sheep grazing was delayed until July 4^th^ to allow measurements of shoot canopy area (cm^2^) as approximated by a circle using the measured diameter of the shadow cast by the shoot canopy on the ground at solar noon, shoot height (cm), basal plant diameter (cm) at the soil surface, and stem number on all plants within the six grazed blocks. After these phenotypic data were collected, sheep were allowed to graze the designated six blocks until 30 August 2012. The use of sheep in grazing experiments was approved and in accordance with guidelines by the animal care committee of College of Ecology and Environmental Science, Inner Mongolia Agricultural University.

### Tissue collection for RNA samples

One week after the sheep were removed from the field plots in 2012, all plants were dug from one randomly selected block of the grazing trial. Equal quantities (fresh weight) of leaf, stem, and root tissues from each plant were individually collected, flash-frozen in liquid nitrogen, and stored separately. Equal quantities of these tissues from the 10 plants of each population were then combined within their respective tissue type to form leaf, stem, and root tissue bulks representing the grazed MF200401 and MF200402 populations. At the same time that the sheep were removed from the grazed plots, the shoots of all plants in the ungrazed block were also cutback to similar height as the grazed plants. The same procedure described for the grazed plants was then used to collect and bulk the leaf, stem and root tissues from the MF200401 plants in the ungrazed block. However, tissues were not collected from the MF200402 population in the ungrazed block because our primary interest was to identify grazing responsive genes in the MF200401 tolerant plants, and genes which were also differentially responsive between the tolerant MF200401 and intolerant MF200402 populations subjected to grazing.

### RNA isolation, validation, and RNA-seq library preparation

RNA was isolated from each of the above tissue bulks, and equal quantities of leaf, stem, and root RNA were pooled within each population and grazing treatment. Therefore, a total of three RNA bulks representing the grazed and non-grazed MF200401 tolerant population and the grazed MF200402 intolerant population were available for transcriptome sequencing analysis.

Total RNA was extracted with TRIzol reagent (Life Technologies, Carlsbad, CA, USA) according to the manufacturer’s instruction and treated with RNase-free DNase I (Takara Biotechnology, Dalian, China). An Agilent 2100 Bioanalyzer (Agilent, Santa Clara, CA, USA) was then utilized to confirm RNA integrity based on a minimum RNA integrated threshold value of eight. Poly(A) mRNA was isolated with oligo-dT beads and then treated with fragmentation buffer. The cleaved RNA fragments were then transcribed into first-strand cDNA using reverse transcriptase and random hexamer primers. This was followed by second strand cDNA synthesis using DNA polymerase I and RNaseH. The double-stranded cDNA was further subjected to end-repair using T4 DNA polymerase, Klenow fragment, and T4 Polynucleotide kinase followed by a single nucleotide “A” base addition using Klenow exo2 polymerase. The cDNAs were then ligated with adapters using T4 DNA ligase. Adaptor ligated fragments were subjected to agarose gel electrophoresis and cDNA fragments within the desired size range (304 ~ 374 bps) were excised from the gel. PCR was performed to amplify these fragments. The quality of the cDNA fragments was validated using an Agilent 2100 Bioanalyzer and a StepOnePlus Real-Time PCR System (Life Technologies, Carlsbad, CA, USA), after which the cDNA library was sequenced using a flow cell HiSeq2000 sequencer (Illumina, San Diego, CA, USA).

### *De novo* transcriptome assembly and functional annotation

Transcriptome *de novo* assembly was carried out with the sequence assembly program ‘Trinity’^87^. Trinity combines three independent software modules – Inchworm, Chrysalis, and Butterfly – applied sequentially to process large volumes of RNA-seq reads. This program partitions the sequence data by constructing many individual de Bruijn graphs, each representing the transcriptional complexity at a given gene or locus. It then processes each graph independently to extract full-length splicing isoforms and to tease apart transcripts derived from paralogous genes. We obtained unique gene sequences from the unigenes using TGI Clustering tools (http://sourceforge.net/projects/tgicl/). In the last step, blastx (E-value < 0.00001) was employed to search for unigene homologs in protein databases including nr, Swiss-Prot, KEGG, and Clusters of Orthologous Group (COG) database. The best results were used to further determine the sequence orientation of unigenes. In case of conflicting results from different databases, a priority order (nr, Swiss-Prot, KEGG and COG) was followed to determine sequence orientation. When a unigene did not align to any of the above databases, ESTScan^88^ was used to predict its coding regions and to determine its sequence orientation. Functional annotation by gene ontology terms (GO; http://www.geneontology.org) was analyzed with the program Blast2go. The COG and KEGG annotations were performed using Blastall^89^.

### Differential unigene expression

All reads which uniquely aligned to a specific assembled transcript were counted using SOAP^90^. Then the FPKM value for each transcript was measured in fragments per kilobase of transcript sequence per million mapped reads. The transcript fold change between two samples was then calculated by the log_2_-ratio of the FPKM values. If the value of FPKM was zero, we used 0.01 instead to calculate the fold change. We applied differential expression analysis^91^ based on the Poisson distribution, as follows. Let *x* and *y* be the mapped clean read counts of the transcript in each sample respectively. Let *N*_*1*_ and *N*_*2*_ be the total numbers of clean reads in the two samples, respectively. Under the null hypothesis of a gene being not differential between two samples, the conditional probability *P* (*y|x*) is


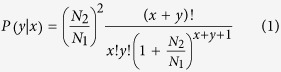


and the accumulative conditional probability Pr (*Y* ≤ *y|x*) is


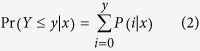


This result assumed a Poisson distribution of the count under the null hypothesis. Under the non-differential null hypothesis, the probability of a specific gene being expressed more differentially than the observed expression level is given by


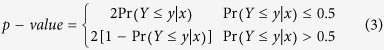


We then computed the q-values to reflect family wise false discovery rate (FDR) to compensate for the multiple testing associated with many simultaneous analyses. In this study, we used FDR ≤0.001 and the absolute value of log2 ratio ≥1 to determine statistically significant differential gene expression.

### Gene ontology functional enrichment analysis

In order to understand the functional ontology categories associated with differentially expressed genes (DEGs), we conducted Gene Ontology^92^ (GO) enrichment analysis, which recognizes the main biological functions that DEGs may exercise by an over-representation strategy. The analysis first counts the numbers of all genes and also DEGs for each GO term in the GO database. Then it performs a hyper-geometric test to find significantly enriched GO terms in DEGs compared to the overall transcriptome background. The statistical significance is calculated by


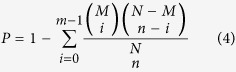


where *N* is the number of all genes with GO annotation, *n* is the number of DEGs in *N*, *M* is the number of all genes that are annotated to certain GO terms, *m* is the number of DEGs in *M*. The calculated p-values were subjected to Bonferroni multiple testing correction. Significance was declared at a threshold of 0.05.

### Pathway enrichment analysis

To explore the underlying molecular mechanisms and biochemical pathways involved in grazing tolerance, we performed pathway enrichment analysis. This process initially utilized the Blastall program to annotate the DEGs against the KEGG database. Subsequently, we adopted the same over-representation strategy used in the GO analysis (Eq. [4]) to carry out KEGG pathway enrichment analysis.

### Real-time RT-PCR analysis

Based on the unigene differential expression analysis results, a subset of 12 transcripts were identified with the following characteristics: each transcript increased in abundance in response to grazing of the tolerant alfalfa plants, and each was differentially expressed in the tolerant and intolerant populations that were subjected to grazing. To validate those results, real-time RT-PCR analysis was subsequently performed on an Opticon II system (MJ Research, Waltham, MA, USA), with SYBR® green reagents (Bio-Rad, Hercules, CA) in a 20 μl reaction volume that contained 250 nM transcript-specific primer pairs (Table 6). The RNA templates for this analysis were derived from three plants each of the tolerant and intolerant populations which had been subjected to grazing and then allowed to recover for one week (i.e. grazing pressure removed). Each plant was dug from the field and leaf, stem, and root tissues were collected as previously described, with the exception that tissues were not bulked over plants. For each plant, total RNA was isolated from each tissue type. Equal quantities of leaf, stem, and root RNA were then pooled within a given plant providing three biological replicates of each population. The total RNA from of each plant was subsequently reverse transcribed and the synthesized cDNA was used as template in RT-PCR with transcript-specific primer pairs. All reactions were replicated twice. Expression of each transcript was normalized using two reference genes, ubiquitin protein ligase 2a (UBL-2a) and actin depolymerizing factor (ADF); both of which have demonstrated stable expression in alfalfa across diverse environmental conditions and plant developmental stages^93^.

### Availability of supporting data

The data discussed in this publication have been deposited in NCBI’s Gene Expression Omnibus^94^ and are accessible through GEO Series accession number GSE50430 (http://www.ncbi.nlm.nih.gov/geo/query/acc.cgi?acc=GSE50430).

## Additional Information

**How to cite this article**: Wang, J. *et al*. Transcriptome responses in alfalfa associated with tolerance to intensive animal grazing. *Sci. Rep*. **6**, 19438; doi: 10.1038/srep19438 (2016).

## Supplementary Material

Supplementary Information

## Figures and Tables

**Table 1:** Phenotypic characteristics of grazing tolerant and intolerant alfalfa plants.

Trait	Grazing Tolerant Plants n = 10		Grazing Intolerant Plants n = 10		Group Difference
	Sample mean	Standard deviation	Sample mean	Standard deviation	P-value
Shoot canopy area (cm^2^)	9669.70	5890.62	602.60	249.45	8.8 × 10^−4^
Basal plant diameter (cm)	13.95	2.26	7.45	1.57	1.3 × 10^−6^
Shoot height (cm)	79.70	9.90	30.80	11.25	6.5 × 10^−9^
Stem number	72.70	14.77	23.00	12.12	2.2 × 10^−7^

All plants were continuously grazed by sheep during 2011. In 2012, sheep grazing was delayed until the fourth of July to allow measurements of the traits listed above. The P-values were computed by the Welch two-sample t-test. Differences in all four traits between the tolerant and intolerant plants are statistically significantly.

**Table 2:** Enriched Gene Ontology (GO) terms associated with grazing responsive genes in a grazing tolerant alfalfa population that were also differentially expressed between tolerant and intolerant populations subjected to grazing.

GO Index	GO Term	Grazing Response P-value	Differential Tolerance P-value
**Cellular Component GO Terms**			
GO:0005840	ribosome	4.00E-40	1.33E-44
GO:0022626	cytosolic ribosome	3.56E-40	3.40E-40
GO:0044445	cytosolic part	3.96E-36	5.15E-37
GO:0030529	ribonucleoprotein complex	4.38E-26	1.09E-33
GO:0044391	ribosomal subunit	3.08E-30	4.66E-30
GO:0015935	small ribosomal subunit	1.78E-15	4.26E-19
GO:0022627	cytosolic small ribosomal subunit	5.19E-12	8.61E-15
GO:0022625	cytosolic large ribosomal subunit	1.87E-15	6.39E-13
GO:0043228	non-membrane-bounded organelle	1.64E-32	8.41E-12
GO:0043232	intracellular non-membrane-bounded organelle	1.64E-32	8.41E-12
GO:0015934	large ribosomal subunit	1.09E-13	8.51E-11
GO:0005730	nucleolus	1.60E-12	4.54E-07
GO:0030312	external encapsulating structure	2.19E-38	2.28E-06
GO:0005618	cell wall	3.37E-39	4.22E-06
GO:0005576	extracellular region	3.82E-25	0.00155
GO:0031981	nuclear lumen	0.00018	0.03452
**Molecular Function GO Terms**			
GO:0003735	structural constituent of ribosome	1.03E-22	2.15E-19
GO:0005198	structural molecule activity	8.77E-23	4.66E-12
GO:0004737	pyruvate decarboxylase activity	0.00268	0.00163
GO:0020037	heme binding	1.33E-08	0.00929
GO:0016702	oxidoreductase activity, acting on single donors with incorporation of molecular oxygen, incorporation of two atoms of oxygen	0.00709	0.01066
GO:0046906	tetrapyrrole binding	1.00E-07	0.01295
GO:0019825	oxygen binding	0.03365	0.01539
**Biological Process GO Terms**			
GO:0006412	Translation	4.60E-13	1.68E-20
GO:0001666	response to hypoxia	0.00678	3.03E-06
GO:0036293	response to decreased oxygen levels	0.00983	5.80E-06
GO:0070482	response to oxygen levels	0.00983	5.80E-06

The GO terms were grouped into cellular component, molecular function, and biological process categories. Within each category the terms were ranked by their differential tolerance P-values, which indicate the statistical significance of differential expression observed between the grazed tolerant and grazed intolerant plants for genes associated with each GO term. Grazing response P-values indicate the statistical significance of differential expression observed between the grazed tolerant and ungrazed tolerant plants for genes associated with each GO term.

**Table 3:** Single nucleotide polymorphism (SNP) in a grazing suppressed gene annotated by three GO biological process terms: response to hypoxia, response to decreased oxygen levels, and response to oxygen levels.

			Tolerant plants				Intolerant plants		Amino acid
			MF200401 (grazed)		MF200401-1 (not grazed)		MF200402 (grazed)		change
Unigene ID	Pos from 5′ end	Base	SNP	Base	SNP	Base	SNP	Base	
*Medicago truncatula isoflavone 2*′*-hydroxylase*									
CL5196.Contig1_All	1604	G	—	G	—	G	K	G:T	3′ UTR

Homologous transcripts of this gene were suppressed by grazing in the MF200401 tolerant plants, and were expressed at even lower levels in the grazed MF200402 intolerant plants. A SNP was reported for transcripts that were monomorphic between the transcriptomes of the grazed and ungrazed tolerant plants, but polymorphic with respect to the transcriptome of the intolerant plants. Pos is the position of the SNP on the assembled transcript from the 5′ end based on sequence data from all samples. Base is the consensus nucleotide observed across all three alfalfa transcriptomes evaluated. SNP is the IUPAC code for nucleotide variation observed at a given position. Amino acid change is obtained by aligning the SNP with coding sequences of known proteins. If the SNP is not located on any coding sequences, it is annotated by either 5′ or 3′ untranslated region (UTR).

**Table 4:** Molecular pathways responsive to grazing and demonstrating differential gene expression between the grazed tolerant and grazed intolerant plants.

Pathway ID	KEGG Pathway Name	Grazing Response		Differential Tolerance	
		P-value	Q-value	P-value	Q-value
ko03010	Ribosome	2.98e-26	6.31e-25	4.58e-52	5.86e-50
ko00940	Phenylpropanoid biosynthesis	1.68e-14	1.95e-13	3.88e-08	1.65e-06
ko00908	Zeatin biosynthesis	0.0846	0.0219	1.05e-06	2.69e-05
ko00945	Stilbenoid, diarylheptanoid and gingerol biosynthesis	3.30e-14	3.50e-13	1.68e-06	3.58e-05
ko00904	Diterpenoid biosynthesis	0.0385	0.0873	2.40e-05	4.39e-04
ko00073	Cutin, suberine and wax biosynthesis	1.51e-04	5.49e-04	0.000142	2.02e-03
ko00040	Pentose and glucuronate interconversions	1.58e-26	4.00e-25	0.000527	6.75e-03
ko00500	Starch and sucrose metabolism	1.08e-13	1.06e-12	0.000608	7.08e-03
ko00909	Sesquiterpenoid and triterpenoid biosynthesis	0.0195	0.0476	0.00107	1.14e-02
ko03020	RNA polymerase	1.50e-03	4.77e-03	0.00191	1.88e-02
ko00511	Other glycan degradation	1.82e-05	7.98e-05	0.00264	2.42e-02
ko00460	Cyanoamino acid metabolism	4.24e-05	1.63e-04	0.00285	2.43e-02
ko00943	Isoflavonoid biosynthesis	3.36e-04	1.16e-03	0.00377	3.02e-02
ko00010	Glycolysis/Gluconeogenesis	2.07e-25	3.28e-24	0.00649	4.89e-02
ko00903	Limonene and pinene degradation	3.76e-12	2.99e-11	0.00886	5.67e-02
ko01110	Biosynthesis of secondary metabolites	3.94e-51	2.50e-49	0.010124	6.17e-02
ko00052	Galactose metabolism	3.03e-03	9.41e-03	0.0129	7.52e-02
ko00906	Carotenoid biosynthesis	1.95e-05	8.25e-05	0.0167	9.32e-02
ko00941	Flavonoid biosynthesis	1.69e-14	1.95e-13	0.0289	1.54e-01
ko00360	Phenylalanine metabolism	0.0182	0.0454	0.0373	1.77e-01
ko00944	Flavone and flavonol biosynthesis	8.45e-16	1.19e-14	0.0447	1.97e-01

*De novo* assembled transcripts were aligned to their corresponding genes in the KEGG pathway database for Medicago. Over-representation of each pathway was determined by the null hypergeometric distribution. Grazing response tests were performed between the tolerant grazed and ungrazed plants and differential tolerance tests were performed between the tolerant grazed and the intolerant grazed plants. The P-values and adjusted Q-values indicate the statistical significance and false discovery rate, respectively, associated with the pathway enrichment analysis.

**Table 5:** Single nucleotide polymorphism (SNP) in five grazing upregulated genes involved in three metabolic pathways.

Unigene ID	Pos from 5′ end	Base	Tolerant plants				Intolerant plants		Amino acid
			MF200401 (grazed)		MF200401-1 (not grazed)		MF200402 (grazed)		change
			SNP	Base	SNP	Base	SNP	Base	
***Pathway ko01110 Biosynthesis of secondary metabolites***:									
*K01850 chorismate mutase* [*EC*:*5.4.99.5*]									
CL10594.Contig2_All	369	A	—	A	—	A	R	G:A	Synonymous
Unigene16098_All	456	A	—	A	R	A:G	W	A:T	Asn**→**Ile (T);Asn**→**Ser (G)
*K15405 cytochrome P450, family 96, subfamily A, polypeptide 15* (*midchain alkane hydroxylase*)									
CL1941.Contig2_All	451	A	W	A:T	—	A	W	A:T	Synonymous
*K12373 hexosaminidase* [*EC*:*3.2.1.52*]									
CL4571.Contig3_All	647	G	R	G:A	R	G:A	R	G:A	Synonymous
*K00869 mevalonate kinase* [*EC*:*2.7.1.36*]									
Unigene11434_All	213	C	Y	C:T	—	C	Y	C:T	5′ UTR
Unigene19167_All	476	T	K	T:G	—	T	K	G:T	3′ UTR
*K13789 geranylgeranyl diphosphate synthase, type II* [*EC*:*2.5.1.1 2.5.1.10 2.5.1.29*]									
Unigene4300_All	174	C	—	C	—	C	M	C:A	Ser**→**Tyr
***Pathway ko00511 Other glycan degradation***:									
*K12373 hexosaminidase* [*EC*:*3.2.1.52*]									
CL4571.Contig3_All	647	G	R	G:A	R	G:A	R	G:A	Synonymous
***Pathway ko00073 Cutin, suberine and wax biosynthesis***:									
*K15405 cytochrome P450, family 96, subfamily A, polypeptide 15* (*midchain alkane hydroxylase*)									
CL1941.Contig2_All	451	A	W	A:T	—	A	W	A:T	Synonymous

Homologous transcripts of these genes were upregulated by grazing in the MF200401 tolerant population and were also more abundantly expressed in the tolerant versus intolerant MF200402 plants, both of which were grazed. A SNP was reported for transcripts that were monomorphic between the transcriptomes of the grazed and ungrazed tolerant plants, but polymorphic with respect to the transcriptome of the intolerant plants. Pos is the position of the SNP on the assembled transcript from the 5′ end based on sequence data from all samples. Base is the consensus nucleotide observed across all three alfalfa transcriptomes evaluated. SNP is the IUPAC code for nucleotide variation observed at a given position. Amino acid change is obtained by aligning the SNP with coding sequences of known proteins. If the SNP is not located on any coding sequences, it is annotated by either 5′ or 3′ untranslated region (UTR).

**Table 6:** Primers used in real-time RT-PCR analysis of 12 grazing upregulated transcripts, as identified from the transcriptome analysis, and two reference genes UBL-2a and ADF.

Transcript ID	Primer-F	Primer-R
Unigene16098_All	5-ATGGCCACAGCAGAGAATGAAT-3	5-tCTTCCAGCCTTAGCTTGAAC-3
CL4571.Contig2_All	5-TTGAATCTATGTCCTACACCA-3	5-CGTCTACTTCTGGCATCACAT-3
Unigene11434_All	5-CACTAGTTACTGCCTCCGAATGT-3	5-GGTGCCTCAGCATTCTCTCAG-3
Unigene19167_All	5-GTCAGTCATGCCACAATAGA-3	5-GACTCCAGTTCAGCAATCACT-3
Unigene4300	5-ATGAGTATGACTCAAACTCCG-3	5-CCACCGACGAGCTCACATGCG-3
CL10594.Contig2_All	5-GCATCAGTGCAGAAGAGAGTA-3	5-GCAACAGGTATTCAACCTGCAC-3
CL10897.Contig2_All	5-CTTCAGCGCTTACATGGCTC-3	5-CCTTAGAGCTTCACATATAGC-3
CL1941.Contig1_All	5-ATCTGATATACTACCTAGTGG-3	5-AAGACGGTACATGTATAATC-3
CL1941.Contig2_All	5-TCTGATATACTACCTAGTGGA-3	5-CTTGTAAGATGGTACATGTAT-3
Unigene26546_All	5-CACTATACATGTACCATCTTA-3	5-TCAAGCCGTGTTTCATGCGA -3
CL4571.Contig3_All	5-AGCAGATAATTGAATCTATGTC-3	5-CAGTTATGCACAACGGTCCG-3
Unigene30838_All	5-TGAATTATTAGCTAGATCCGA-3	5-TGGCAAATCTCATCACCGTCG-3
UBL-2a	5-CCAAACCCAAACTCACCAG -3	5-AGCAGTCCAACTCTGCTCAAC -3
ADF	5-GCATCTGGTATGGCAGTCC-3	5-GCACTCATCAGCAGGAAGG-3
