# Supplementary material for: Transcriptome responses in alfalfa associated with tolerance to intensive animal grazing
Source: Sci Rep. 2016 Jan 14;6:19438. doi: 10.1038/srep19438 (PMC4725929; doi:10.1038/srep19438)
Supplement: Supplementary Information [file srep19438-s1.pdf]

SUPPLEMENTARY INFORMATION

Transcriptome responses in alfalfa associated with tolerance to intensive animal grazing

Junjie Wang[1,*], Yan Zhao[1], Ian Ray[2], Mingzhou Song[3]

1 College of Ecology and Environmental Science, Inner Mongolia Agricultural University, Hohhot, China
2 Department of Plant and Environmental Sciences, New Mexico State University, Las Cruces, NM, USA
3 Department of Computer Science, New Mexico State University, Las Cruces, NM, USA

* Corresponding author. Email: jjw62@163.com

Supplementary Table S1. **Assembled and annotated transcriptomes from grazed and ungrazed plants of a grazing tolerant alfalfa population (MF200401 and MF200401-1, respectively) and an intolerant population that was grazed (MF200402).**

Samples	Total Raw Reads	Total Clean Reads	Total Clean Nucleotides (nt)	Q20 percentage	N percentage	GC percentage
Tolerant & grazed MF200401	52,109,802	48,225,878	4,340,329,020	98.32	0.01	42.95
Intolerant & grazed MF200402	52,238,400	48,024,330	4,322,189,700	98.29	0.01	42.54
Tolerant & not grazed MF200401-1	52,969,656	49,174,768	4,425,729,120	98.33	0.01	44.22

The number of total clean nucleotides is the total number of nucleotides on all clean paired-end reads. Q20 percentage is defined as the percentage of nucleotides with quality value larger than 20 in reads. N percentage is defined as the percentage of unknown nucleotides in clean reads.

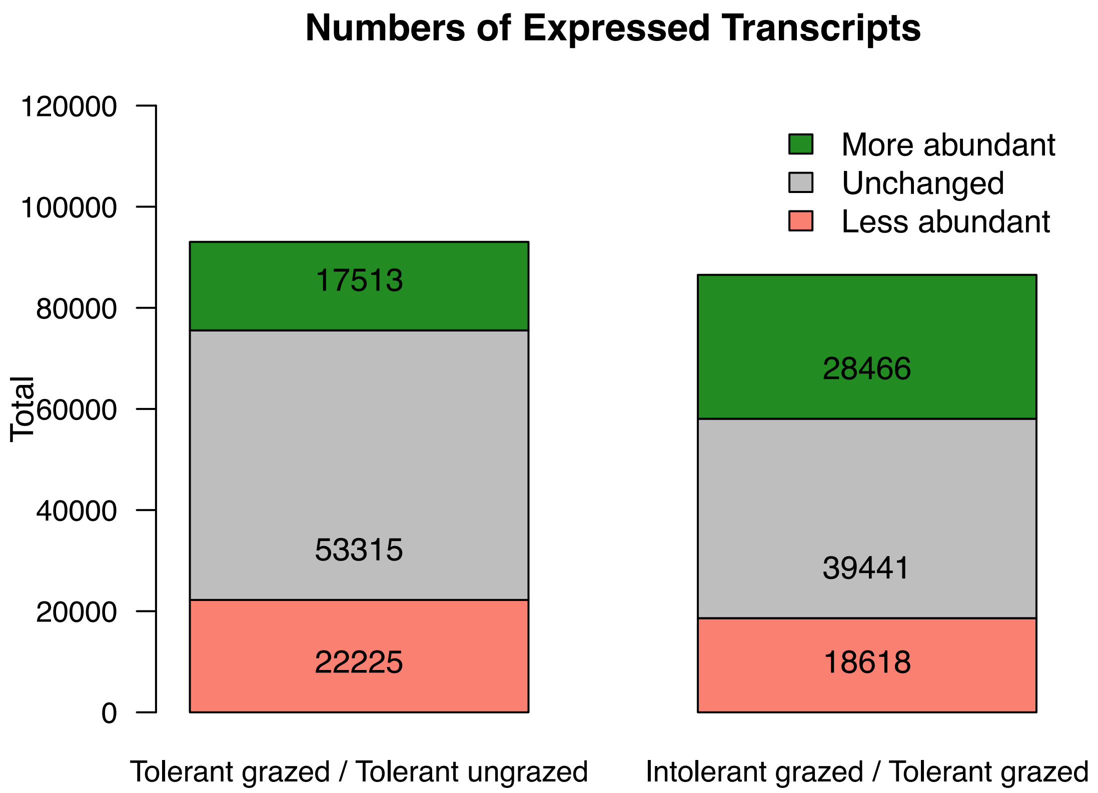

Supplementary Figure S1. Comparative analysis of transcript abundance in three alfalfa transcriptomes. The left bar represents the number of transcripts that were more abundant (17,513 in green), unchanged (53,315 in gray), and less abundant (22,225 in red) in the grazed tolerant plants as compared to the ungrazed tolerant plants. The right bar represents the number of genes with transcripts that were more abundant (28,466 in green), unchanged (39,441 in gray), and less abundant (18,618 in red) in the grazed intolerant plants as compared to the grazed tolerant plants. The statistical significance of all differentially expressed transcripts was set at $P \leq 0.05$, adjusted for family-wise false discovery rate by the Benjamini-Hochberg method.

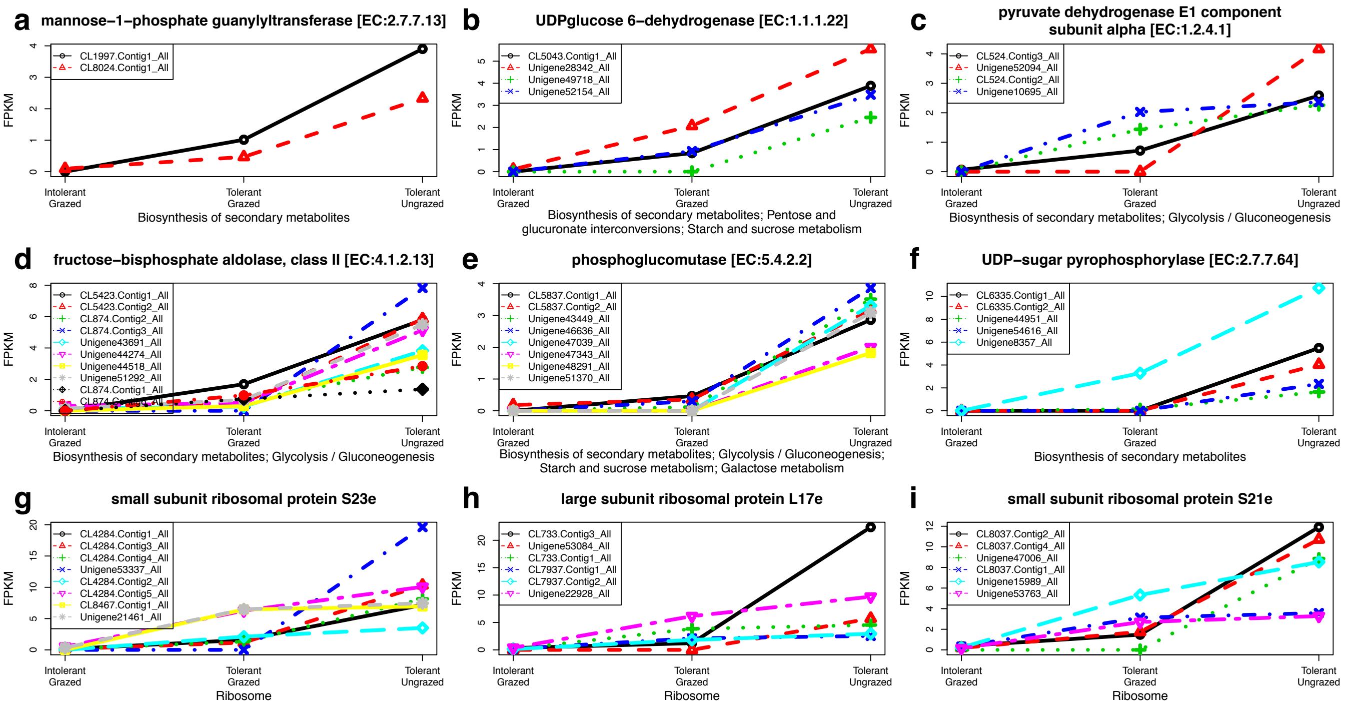

Supplementary Figure S2. Expression of grazing suppressed genes from grazing responsive pathways that were differentially expressed between grazing tolerant and intolerant alfalfa plants. Fifty-three transcript isoforms from 9 genes in grazing tolerant plants were suppressed in response to grazing. However, these genes were expressed at higher levels in the grazed tolerant plants than the grazed intolerant plants. The horizontal axis identifies three transcriptomes that were evaluated: tolerant plants not grazed, tolerant plants grazed, and intolerant plants grazed. The vertical axis, fragments per kilobase of transcript per million mapped reads (FPKM), represents normalized read abundance of homologous transcripts of a given gene. Pathways associated with each gene are also provided. Note: Figures 3a and 3b initially suggested that EC 5.1.3.3 (Aldose Epimerase) was suppressed by grazing of tolerant plants, and was more abundantly expressed in the tolerant versus intolerant plants, both of which were grazed. However, additional analysis of the gene products indicated that different EC 5.1.3.3 transcripts were associated with the Figure 3a and 3b results. Consequently, no consistent suppression pattern was identified for a given transcript, and therefore, this gene was not represented in the current figure.

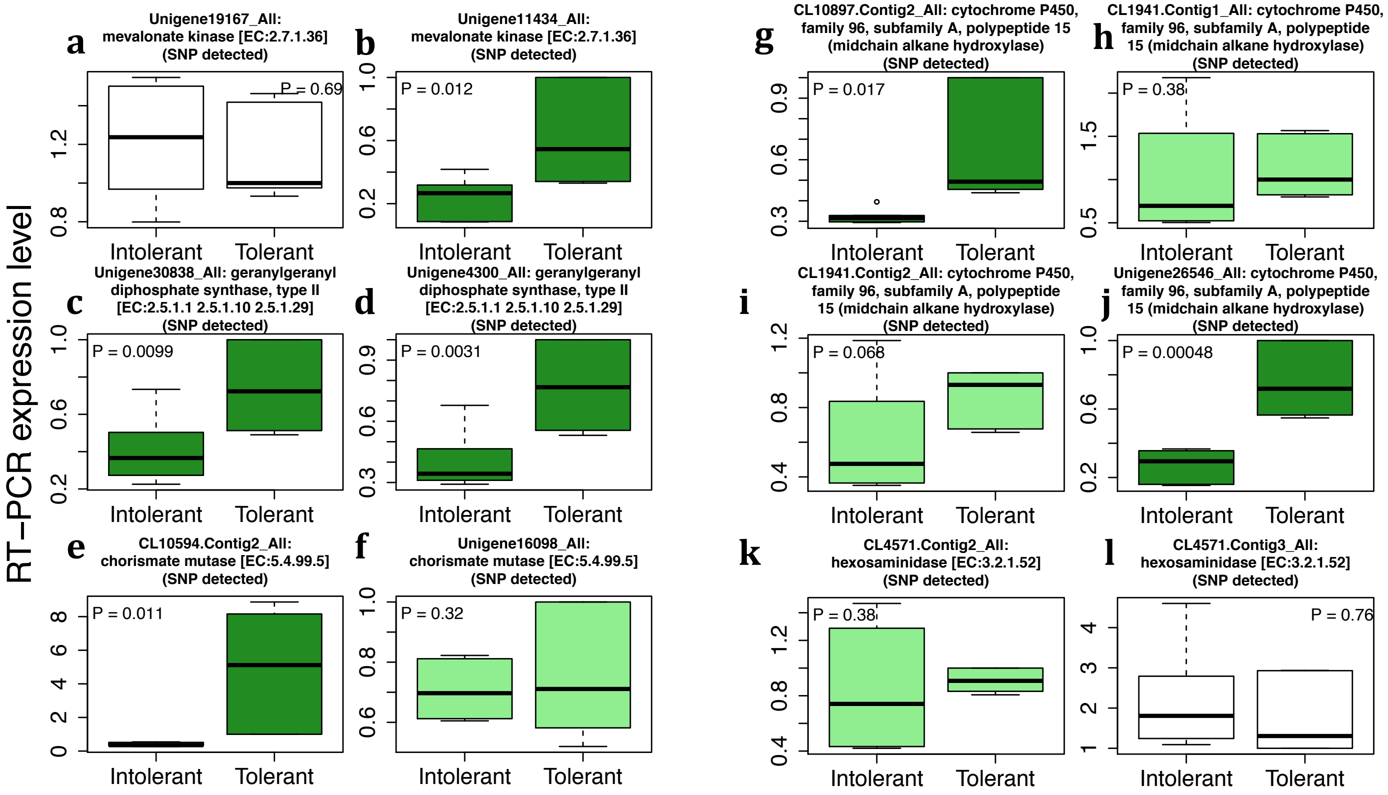

Supplementary Figure S3. Independent validation of grazing-upregulated transcripts by RT-PCR. Twelve transcripts from five grazing-upregulated transcript isoform groups were selected for RT-PCR expression quantification to validate transcriptome analysis results. Three biological replicates consisting of three grazing tolerant and three intolerant alfalfa plants, and two technical replicates of each plant, were used in the RT-PCR analysis. Transcript identities and their encoded proteins are given as plot titles. Box plots indicate the median and range of gene expression observed for each of the 12 transcripts among the tolerant and intolerant plants. The *P*-value of *t* tests for differential expression between the tolerant and intolerant alfalfa plants for each transcript is given in each plot. Six of the twelve transcripts (dark green box plots) confirm the RNA-seq results, which indicated that these transcripts were expressed at significantly higher levels in the tolerant versus intolerant plants that were grazed. Four of the remaining transcripts (light green box plots) suggest that they were also more abundant in the tolerant versus intolerant plants. Two transcripts in white box plots are inconsistent with the transcriptome data. **(a,b)** Two transcript isoforms of mevalonate kinase. **(c,d)** Two transcript isoforms of geranylgeranyl diphosphate synthase, type II. **(e,f)** Two transcript isoforms of chorismate mutase. **(g-j)** Four transcript isoforms of a protein cytochrome P450, family 96, subfamily A, polypeptide 15 (midchain alkane hydroxylase). The circle in (g) indicates an outlier. **(k,l)** Two transcript isoforms of hexosaminidase.

a

b

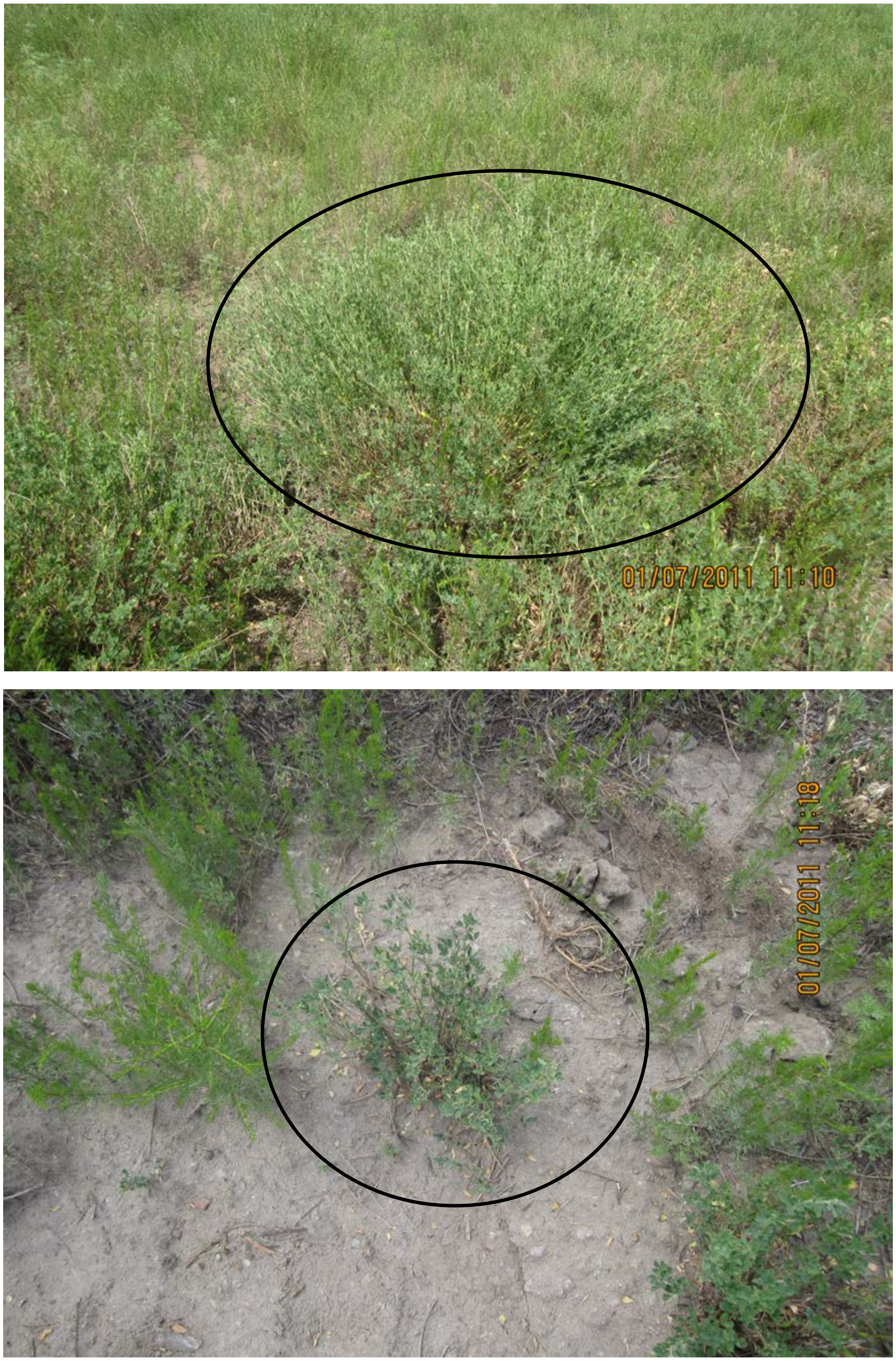

Supplementary Figure S4. Alfalfa M. sativa plants after continuous grazing in 2010 but before grazing in 2011. Both photos were taken on July 1, 2011. **(a)** Grazing tolerant alfalfa plant (MF200401). **(b)** Grazing intolerant plant (MF200402).
